# Effectiveness of cloth face masks to prevent viral spread: a meta-analysis

**DOI:** 10.1093/pubmed/fdad205

**Published:** 2023-11-02

**Authors:** Elisabeth L Zeilinger, Nadine Brunevskaya, Jana Wurzer, Sandra Oberleiter, Jonathan Fries, Amelie Fuchs, Alma Herscovici, Lea Kum, Eva K Masel, Jakob Pietschnig

**Affiliations:** Division of Palliative Medicine, Department of Medicine I, Medical University of Vienna, A-1090 Vienna, Austria; Academy for Ageing Research, Haus der Barmherzigkeit, A-1160 Vienna, Austria; Department of Clinical and Health Psychology, Faculty of Psychology, University of Vienna, A-1010 Vienna, Austria; Division of Palliative Medicine, Department of Medicine I, Medical University of Vienna, A-1090 Vienna, Austria; Department of Clinical and Health Psychology, Faculty of Psychology, University of Vienna, A-1010 Vienna, Austria; Division of Hematology and Hemostaseology, Department of Medicine I, Medical University of Vienna, A-1090 Vienna, Austria; Department of Developmental and Educational Psychology, Faculty of Psychology, University of Vienna, A-1010 Vienna, Austria; Department of Developmental and Educational Psychology, Faculty of Psychology, University of Vienna, A-1010 Vienna, Austria; Division of Hematology and Hemostaseology, Department of Medicine I, Medical University of Vienna, A-1090 Vienna, Austria; Division of Hematology and Hemostaseology, Department of Medicine I, Medical University of Vienna, A-1090 Vienna, Austria; Division of Palliative Medicine, Department of Medicine I, Medical University of Vienna, A-1090 Vienna, Austria; Division of Palliative Medicine, Department of Medicine I, Medical University of Vienna, A-1090 Vienna, Austria; Department of Developmental and Educational Psychology, Faculty of Psychology, University of Vienna, A-1010 Vienna, Austria

**Keywords:** COVID-19, face masks, filtration efficiency, non-pharmaceutical intervention

## Abstract

**Background:**

The effectiveness of cloth face masks to prevent viral spread has not yet been conclusively established. In this meta-analysis, we evaluate their effectiveness in comparison to standard medical/surgical and N95-typed masks against viral spread.

**Methods:**

We identified literature through a systematic search in three databases and meta-analytically synthesized relevant studies by means of random-effects as well as multilevel modelling.

**Results:**

Twelve studies comprising *k* = 28 effect sizes (*N* = 338) were included. Medical/surgical and N95-typed masks outperformed cloth masks, yielding a large effect (*g* = 1.40). This effect remained robust when data were grouped according to comparisons with medical/surgical masks (*g* = 1.25) and N95-typed masks (*g* = 1.29). However, effects were differentiated according to mask fit, indicating reversals of signs when cloth mask effects were compared with ill-fitting medical/surgical and N95-typed masks (*g*s = −12.50 and − 10.90, respectively).

**Conclusions:**

Cloth face masks were found to have significantly poorer filtering performance than medical/surgical masks and N95 masks, but only if non-cloth masks were properly fitted. Our results illustrate the necessity of using well-fitting medical/surgical or N95-typed masks to prevent viral spread, although some allowance should be made in circumstances where higher compliance with cloth mask mandates are expected.

## Introduction

The COVID-19 (Coronavirus Disease 2019) pandemic caused by SARS-CoV-2 (Severe Acute Respiratory Syndrome Coronavirus 2) has significant implications for public health and health policies worldwide and has already led to many deaths, especially in the elderly and people with pre-existing medical conditions. Although different vaccines have become available by now, global vaccination rates are currently insufficient to yield expectable population immunity any time soon ^1^ and it remains unclear if immunity will be achieved at all.^2^ Current global patterns of infection rates suggest that the COVID-19 pandemic is not over yet.^3^

The SARS-CoV-2 pathogen is mainly transmitted via respiratory droplet infection and aerosols. When speaking, humans exhale ~20 000 droplets per second, mainly depending on the volume of speech.^4^^,^^5^ Exposure to these droplets is largest within 1.5–2 m of the speaker.^6^ Wearing face masks can contribute to substantially reducing this exposure.^7^ Obviously, effects of mask wearing are not limited to protection against COVID-19 infections but have similar effects in regard to the exposure to other pathogenic aerosols. However, particularly in the general public, the use of face masks in healthy people is still discussed controversially. In addition to their potential benefits, face masks can also have negative effects, such as causing a false sense of security which may lead to neglecting other protective measures (e.g. use of hand sanitizer or distancing rules) or health problems caused by restricted air circulation.^8^

To prevent spreading of SARS-CoV-2, a number of countermeasures have been formally implemented in many countries.^9^ The most important prevention measures were distancing rules as well as, especially in circumstances or locations where distancing was difficult to implement, the additional wearing of face masks and hand hygiene measures. Such recommendations have been established by the European Centre for Disease Prevention and Control and are particularly aimed at reducing the spread of SARS-CoV-2 by pre-symptomatic or asymptomatic infected people.^10^ Most mask wearing mandates to date relate to the use in closed and confined spaces, such as public transportation, health facilities, or educational institutions. However, wearing masks can also prevent infections outdoors and must be considered useful when large numbers of people congregate in events such as in mass gatherings, because it is typically unlikely that a safe distance can be maintained between people throughout these events.^11^^,^^12^

Cloth face masks, and especially homemade masks, are a common and readily available measure to protect oneself and others from infection. Especially, at the beginning of the pandemic, people in several countries were encouraged to wear cloth masks instead of surgical masks to avoid a shortage of surgical masks for medical personnel.^13^ The considerable use of cloth face masks led to different research efforts examining their effectiveness in preventing SARS-CoV-2 infections. However, the effectiveness of cloth face masks in comparison to standard surgical masks or N95-typed masks in preventing viral infections is not yet fully understood, and studies report mixed results (e.g. ^14^^,^^15^).

Here, we present the first formal meta-analytical synthesis of the effectiveness of cloth face masks in reducing SARS-CoV-2 transmission compared to standard medical/surgical or N95-typed masks. We can therefore provide evidence-based recommendations regarding the potential usefulness of wearing cloth face masks.

## Material and methods

### Literature search

Since a narrative systematic review on cloth face masks effectiveness had been previously published,^13^ we used this study as starting point for a cited reference search in three databases (Google Scholar, ISI Web of Science and SCOPUS). This strategy was based on the assumption that any novel primary study on the effectiveness of cloth masks can be deemed likely to cite an existing review on the topic. Abstracts and titles of 82 records were screened, and subsequently full texts of 22 studies were assessed, out of which five met inclusion criteria. Second, reference lists of these 22 studies were screened for additional potentially relevant studies. Another seven studies that met our inclusion criteria were identified in this way, leading to a total of 12 studies for formal analysis.

Duplicates were removed by one researcher. All other steps in the literature search, including abstract screening and full text evaluations were performed independently by at least two out of four trained researchers (AF, AH, NB and JW). Agreement in abstract screening and full text screening was 88.7 and 100%, respectively. Discrepancies were resolved by including a further researcher in discussion (ELZ). Examples for study exclusion reasons were the use of machine learning models instead of provision of empirical data,^16^ or no data about cloth face masks.^17^ A PRISMA-flowchart of study inclusion is provided in Supplementary Fig. S1, see online supplementary material for a colour version of this figure.

### Inclusion criteria

Studies had to fulfil the following three criteria to be included in our analyses. First, they had to report indices for mask performance (e.g. filtration efficiency, particle penetration) for cotton cloth masks (in cases where multiple variants of cotton cloth masks were compared, results of single-layered masks with largest thread counts per area were preferred over other cotton materials) as well as either medical/surgical masks, N95-typed masks (i.e. masks that conformed to the N95 standard, such as FFP1, FFP2 or FFP3-masks), or both. Second, mask performance had to have been tested based on a minimum of three observations (i.e. multiple testing of a single mask, single testing on multiple masks of the same type or a combination of both) and the exact number of these observations had to have been provided to allow standard meta-analytical weighting of effect sizes according to assessment precision. Third, sufficient statistical parameters had to be reported to allow a calculation of standardized mean differences (Hedges *g*).

### Coding

All studies were coded twice independently by two trained researchers (NB, JW). Studies were coded into categories according to mask fit (medical/surgical masks and N95-typed masks fitted with or without gap), mask type (cotton cloth versus medical/surgical versus N95-typed), measured particles (e.g. aerosols, viral RNA), outcome type (e.g. filtration efficiency, particle penetration) and statistical parameters were assessed. Moreover, potential data dependencies between effect sizes were recorded to allow modelling by means of a multilevel approach. Coding discrepancies were resolved through discussion with two independent researchers (SO, JP).

### Statistical analyses

We calculated standardized mean differences with small sample size correction (i.e. Hedges *g*; positive values indicate better performance of non-cloth masks). If results for more than one mask of the same type had been reported separately within primary studies (e.g. if filtration efficiencies for two cotton cloth masks across three time points had been reported separately), means and standard deviations were averaged to obtain a group-wise value before effect size calculation.

Primary study effect sizes were synthesized into meta-analytical summary effects by means of random-effects analyses. We first synthesized effects of comparisons between cotton cloth masks and other masks (i.e. both medical/surgical and N95-typed) in all available data. Subsequently, we carried out subgroup analyses for performance comparisons between (i) cloth masks and medical/surgical masks as well as (ii) cloth masks and N95-typed masks. Due to partial statistical dependencies of several effect sizes, we applied multilevel maximum likelihood estimations which take dependencies into account.

In another meta-analytical subset, we synthesized only those (independent) effect sizes that were based on comparisons of cloth with non-cloth-typed masks that had either been fitted appropriately (i.e. representing a scenario where there was no gap between the masks and the face) or inappropriately (i.e. a scenario where there was a gap).

To evaluate effect robustness and influences of potential leverage points, sensitivity analyses were conducted. In this vein, we ran leave-one-out analyses by iteratively omitting single effect sizes in each turn of a meta-analytical summary effect estimation of a given data (sub-)set. If the resulting summary effect estimates remain comparatively unaffected whilst single effects are left out, this is indicative of summary effect stability against leverage point influences.

Instead of formal null-hypothesis significance testing, we interpret our findings in terms of the well-established effect size thresholds according to Cohen^18^ with absolute standardized mean differences of *g* = 0.2, 0.5 and 0.8 representing the lower thresholds of small, moderate, and large effects, respectively. All analyses were conducted by means of the open source software R^19^ and the package metafor.^20^

### Final sample

In all, we included results from 12 studies comprising *k* = 28 effect sizes (*N* = 338) in our meta-analysis. In our subsets, cloth mask versus medical/surgical mask analyses consisted of 18 effects (*n* = 219) whilst those of cloth masks versus N95-typed masks consisted of ten effects (*n* = 119). A total of five effect sizes were available from studies that had investigated performance of appropriately versus inappropriately fitted non-cloth masks (two without and with gap, respectively, for comparisons with medical/surgical masks and three for those with N95-typed masks, respectively). Primary study characteristics are summarized in Supplementary Table S1, see online supplementary material for a colour version of this figure. References of included studies are listed in Supplementary File S1, see online supplementary material for a colour version of this figure.

## Results

**Fig. 1:**
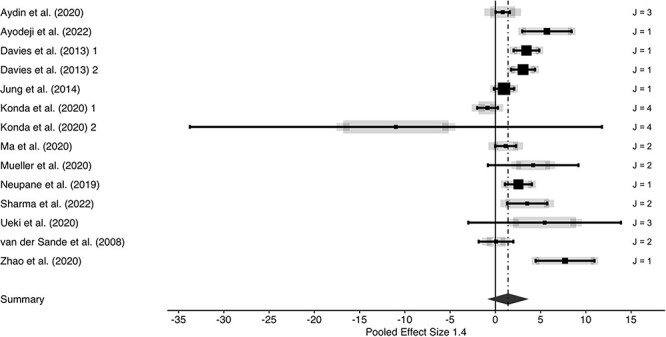
Forest plot of three-level meta-analysis for cotton cloth masks versus any other masks. Positive effect sizes indicate performance advantages of non-cotton cloth masks. Row entries represent independent effects (J indicates number of dependent effects within rows; grey lines are indicative of between-effects variabilities of dependent effects) with symbol size showing study precision (larger symbols represent larger study weights). Whiskers represent 95% confidence intervals. Summary effect and associated 95% confidence interval is represented by a diamond.

**Fig. 2:**
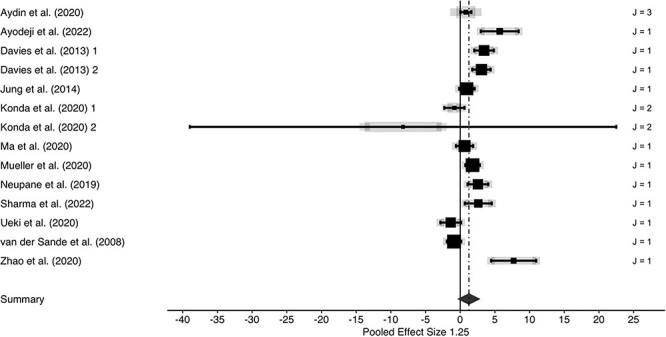
Forest plot of three-level meta-analysis for cotton cloth masks versus medical/surgical masks. Positive effect sizes indicate performance advantages of non-cotton cloth masks. Row entries represent independent effects (J indicates number of dependent effects within rows; grey lines are indicative of between-effects variabilities of dependent effects) with symbol size showing study precision (larger symbols represent higher study weights). Whiskers represent 95% confidence intervals. Summary effect and associated 95% confidence interval is represented by a diamond.

**Fig. 3:**
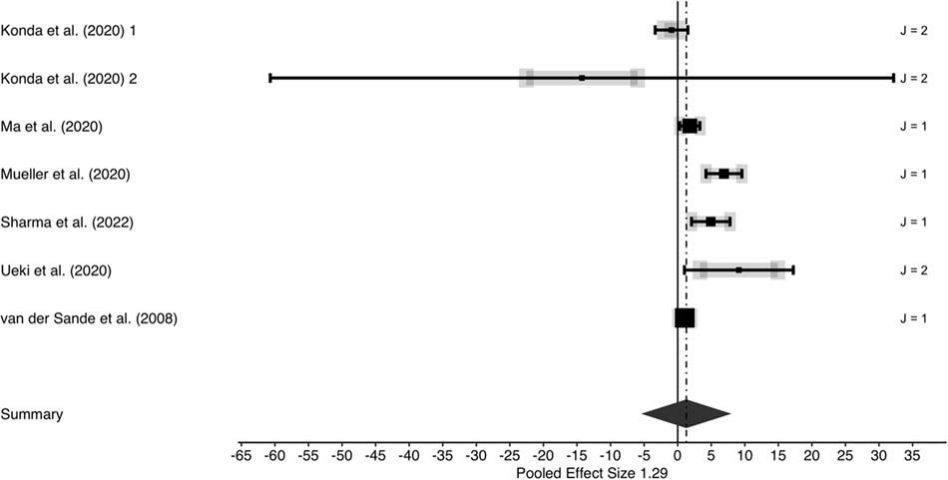
Forest plot of three-level meta-analysis for cotton cloth masks versus N95-typed masks. Positive effect sizes indicate performance advantages of non-cotton cloth masks. Row entries represent independent effects (J indicates number of dependent effects within rows; grey lines are indicative of between-effects variabilities of dependent effects) with symbol size showing study precision (larger symbols represent higher study weights). Whiskers represent 95% confidence intervals. Summary effect and associated 95% confidence interval is represented by a diamond.

We observed substantial performance differences favouring medical/surgical as well as N95-typed masks over cotton cloth masks, yielding a large effect (*g* = 1.40; Fig. 1). The performance advantage of non-cloth masks remained robust when data were grouped according to comparisons of cloth masks versus medical/surgical masks (*g* = 1.25; Fig. 2) and versus N95-typed masks (*g* = 1.29; Fig. 3). However, between-studies heterogeneities were large (all *I*^2^-values exceeded 90%), indicating substantial influences of unobserved study heterogeneity.

Examinations of effects accounting for mask fit indicated considerable performance increases of medical/surgical masks and even larger ones for N95-typed masks when they were fitted appropriately (*g*s = 3.41 and 7.11 when compared with cloth masks, respectively). However, when non-cloth masks were fitted inappropriately, the observed sign changed direction, indicating substantial performance advantages of cloth masks over medical/surgical and N95-typed masks (*g*s = −12.50 and − 10.90, respectively). This means that although medical/surgical and N95-typed masks generally outperform cotton cloth masks, they are considerably less effective than cloth if not worn properly. Numerical results are summarized in Table 1. Of note, all observed summary effects were large in terms of strength, regardless of the observed effect direction.

**Table 1:** Summary effects of cotton cloth masks vs. any other, medical/surgical, or N95-typed masks based on (multi-level) random-effects meta-analyses.

	*k* (level 3)	*n*	*I* ^2^	Hedges *g*	*LCI*	*UCI*
		Cloth masks vs. other masks				
Overall (2-level analysis)	28	338	98.42	1.36	−0.92	3.63
Overall (3-level analysis)	28 (14)	338	98.25	1.40	−0.76	3.56
		Cloth masks vs. medical/surgical masks				
Overall (2-level analysis)	18	219	96.75	1.21	−0.66	3.07
Overall (3-level analysis)	18 (14)	219	96.18	1.25	−0.47	2.97
Cloth masks vs. medical/surgical masks without gap	2	32	96.14	3.41	−3.74	10.57
Cloth masks vs. medical/surgical masks with gap	2	32	96.31	−12.50	−34.61	9.61
		Cloth masks vs. N95-typed masks				
Overall (2-level analysis)	10	119	99.39	1.17	−6.10	8.43
Overall (3-level analysis)	10 (7)	119	99.26	1.29	−5.36	7.94
Cloth masks vs. N95-typed masks without gap	3	38	94.33	7.11	−0.75	14.96
Cloth masks vs. N95-typed masks with gap	3	38	99.35	−10.90	−36.80	15.00

Note. *I^2^* = percentage of variability due to variability of true effects; *LCI* = lower bound of 95% confidence interval; *UCI* = upper bound of 95% confidence interval.

Our sensitivity analyses indicated on the whole no substantial influences of leverage points on our overall analyses or when the analyses were grouped according to comparisons with medical/surgical and N95-typed masks. However, analyses of comparisons according to mask fit (i.e. without and with gap) were rather volatile, thus indicating effect instability which can be attributed to the low number of available primary effect sizes (Supplementary Fig. S2, see online supplementary material for a colour version of this figure.).

## Discussion

The present study investigated the effectiveness of cloth face masks in comparison to standard medical/surgical masks and N95-typed masks for preventing infection with SARS-CoV-2. We found that cloth face masks perform poorly when compared to medical/surgical masks or N95-typed masks, with the latter two types yielding advantages in filtration efficiency of more than a standard deviation. Importantly, these effects were differentiated according to mask fit of the non-cloth masks.

Specifically, signs of mean differences reversed when non-cloth masks were not fitted perfectly (i.e. without a gap), yielding cloth mask advantages of ~12.5 and 11.0 standard deviations compared to medical/surgical and N95-typed masks, respectively. This is highly relevant as it might be argued that individuals may be more willing to wear tight-fitting cloth masks as opposed to medical/surgical or N95-typed ones due to the better breathability of cloth masks.^21^ In fact, some evidence suggests that particularly N95-typed masks are perceived to be considerably less comfortable to wear compared to medical/surgical or cloth masks.^22^

Data indicate that mask wearing compliance is larger for cloth masks than for medical masks, particularly in low-to-middle-income countries, although not all available studies showed entirely equivocal results.^23^ One advantage of (self-made) cloth face masks is the potential of a customized design and an optimal fit to the individual face shape. Following a reasonably simple sewing protocol, self-made cloth masks can be customized to fit their wearers individually and thus also achieve higher filtration efficiency than non-customized masks.^24^

Consequently, it seems reasonable that mandating wearing cloth masks may be justified when this can be expected to yield higher compliance rates than requirements of wearing medical/surgical or N95-typed masks. This could lead to lower infection rates due to (i) higher compliance with the mask mandate in general, (ii) better mask fit due to individually customized designs and (iii) more correctly worn masks because of better breathability and consequently better comfort of cloth masks.

The effectiveness of mitigation measures is strongly dependent on compliance and the willingness of individuals to follow these measures adequately.^25^^,^^26^ The most important perceived disadvantage that reduces compliance of wearing face masks is a general discomfort and breathing problems.^27^^,^^28^ Although CO_2_ concentrations found in all types of masks had no toxicological effects, the observed concentrations can easily cause symptoms such as concentration difficulties, headaches, or fatigue.^29^ These effects can discourage people from wearing face masks. In cloth face masks, breathability depends on the kind of fabric and the number of layers used, with more layers providing higher filtration efficiency but less breathability.^21^^,^^30^ Our analysis focused on single-layer cloth face masks, which was the most frequently tested type in the available studies. When focusing on multi-layered masks, cloth masks might perform better than our analysis suggests, meaning that our present assessment of cloth masks represents a conservative estimate. However, it is highly questionable whether they would outperform well-fitting standard N95 masks or medical masks given the large effect sizes found in the present study. Moreover, because more layers impair breathability, multi-layered cloth masks would come at the cost of increasing discomfort and therefore arguably lead to less compliance.

An established public health tool that had initially been conceived for injury prevention is the Haddon’s matrix, which states that every accident is affected by host, agent (e.g. infectious disease) and environment. The Haddon’s matrix has recently been modified to encompass COVID-19^31^ and includes the adoption of precautionary measures among a variety of other factors in pandemic control. Therefore, the use of face masks, preferably adequately fitting medical/surgical masks and N95 masks, or at the very minimum cloth masks, can be considered as one of several effective measures to protect against the spread of COVID-19 and other communicable diseases. With the present research, we underline the importance of this measure and provide evidence-based, straightforward recommendations for the most effective mask types.

### Limitations

To our knowledge, the present study is the first formal meta-analysis on the effectiveness of cloth face masks compared to medical/surgical and N95-typed masks in relation to viral spread. However, our search strategy was limited to primarily English-language databases, thus precluding inclusion of most non-English publications which may have led to a language bias in the included studies. However, although we cannot entirely rule out the possibility of a resulting confounding effect, there is no tangible reason to suspect that non-English publications should have yielded any systematically different results compared to the presently included studies. In addition, we primarily targeted single-layered cloth face masks and did not conduct separate analyses for different types of cloth face masks due to infrequent reporting within primary studies. Therefore, we cannot rule out that the presently observed differences may be further differentiated according to unobserved heterogeneity. This idea is further supported by the large between-studies heterogeneity which remained substantial even when effect sizes were grouped according to different assessments. However, assessments by means of both standard as well as multilevel modelling yielded virtually identical results, thus indicating robustness of our observed summary effect estimates.

## Conclusions

The present meta-analysis shows that medical/surgical as well as N95-typed masks generally outperform cloth masks. These differences in effectiveness are large, yielding advantages of more than one standard deviation in favour of non-cloth masks. However, this effect appears to be differentiated according to mask fit, indicating that correctly worn cloth masks are more effective than incorrectly worn non-cloth masks. In all, mask mandates should ideally prioritize correctly worn N95-typed or medical/surgical masks, with some allowance being made for circumstances where higher compliance with cloth mask mandates can be expected.

## Funding

This research did not receive any specific grant from funding agencies in the public, commercial, or not-for-profit sectors.

## Conflict of Interests

None.

## Author’s contributions

ELZ was involved conceptualization, data curation, investigation, methodology, project administration, writing—original draft. NB and JW contributed to data curation, investigation, methodology, writing—review & editing. SO undertook data curation, visualization, writing—review & editing. JF performed the formal analysis, writing—review & editing. AF and AH were involved in data curation, writing—review & editing. AH performed data curation, writing—review & editing. LK and EKM undertook investigation, writing—review & editing. JP was involved in conceptualization, investigation, methodology, project administration, supervision, formal analysis, writing—original draft. All authors approved the final manuscript.

## Ethical approval

Due to the nature of the study as a meta-analysis, no ethical approval was needed.

## Data availability

The data that support the findings of this study are available from the corresponding author (ELZ) upon reasonable request.

## Supplementary Material

Supplementary_File_fdad205

## Notes

**Elisabeth L. Zeilinger,** Clinical Psychologist and Health Psychologist

**Nadine Brunevskaya,** Psychology Student

**Jana Wurzer,** Psychology Student

**Sandra Oberleiter,** Psychologist

**Jonathan Fries,** Psychologist

**Amelie Fuchs,** Psychology Student

**Alma Herscovici,** Psychology Student

**Lea Kum,** Medical Doctor

**Eva K. Masel,** Medical Doctor, Professor of Palliative Medicine

**Jakob Pietschnig,** Psychologist, Professor of Psychology
