# Supplementary material for: Effectiveness of cloth face masks to prevent viral spread: a meta-analysis
Source: J Public Health (Oxf). 2023 Nov 2;46(1):e84–90. doi: 10.1093/pubmed/fdad205 (PMC10901268; doi:10.1093/pubmed/fdad205)
Supplement: Supplementary_File_fdad205 [file supplementary_file_fdad205.pdf]

Supplementary material to the paper

"Effectiveness of cloth face masks to prevent viral spread: a meta-analysis"

Table of contents

Supplementary Table 1 2
Supplementary Figure 1 4
Supplementary Figure 2. Leave-one-out analyses for meta-analytical data (sub-)sets. 5
Supplementary File S1. References of papers included in analysis 6

Supplementary Table 1.

Characteristics of included studies.

Study	Country	Comparisons (mask types)	Non-cloth mask fit	Outcome	Particle type	*n* (cloth masks/ other masks)	Hedges *g*	Standard error
Aydin et al. (2020) [a]	USA	cotton cloth vs. medical/surgical	-	blocking efficiency	aerosols	3/3	0.53	0.67
	USA	cotton cloth vs. medical/surgical	-	blocking efficiency	aerosols	3/3	0.91	0.70
	USA	cotton cloth vs. medical/surgical	-	blocking efficiency	aerosols	3/3	1.03	0.72
Ayodeji et al. (2022)	USA	cotton cloth vs. medical/surgical	-	filtration efficiency	aerosols	5/5	5.70	1.40
Davies et al. (2013)	England	cotton cloth vs. medical/surgical	-	filtration efficiency	B atropheus	9/9	3.44	0.73
Davies et al. (2013)	England	cotton cloth vs. medical/surgical	-	filtration efficiency	bacteriophage MS 2	9/9	3.06	0.68
Jung et al. (2014)	Taiwan	cotton cloth vs. medical/surgical	-	penetration	aerosols	5/7	0.96	0.57
Konda et al. (2020) [b]	USA	cotton cloth vs. N95-typed	no gap	filtration efficiency	aerosols	8/8	0.29	0.48
	USA	cotton cloth vs. N95-typed	gap	filtration efficiency	aerosols	8/8	-2.19	0.61
	USA	cotton cloth vs. medical/surgical	no gap	filtration efficiency	aerosols	8/8	-0.13	0.47
	USA	cotton cloth vs. medical/surgical	gap	filtration efficiency	aerosols	8/8	-1.61	0.55
Konda et al. (2020) [c]	USA	cotton cloth vs. N95-typed	no gap	filtration efficiency	aerosols	8/8	8.97	1.65
	USA	cotton cloth vs. N95-typed	gap	filtration efficiency	aerosols	8/8	-38.42	6.81
	USA	cotton cloth vs. medical/surgical	no gap	filtration efficiency	aerosols	8/8	7.18	1.35
	USA	cotton cloth vs. medical/surgical	gap	filtration efficiency	aerosols	8/8	-24.19	4.30

Study	Country	Comparisons (mask types)	Non-cloth mask fit	Outcome	Particle type	*n* (cloth masks/ other masks)	Hedges *g*	Standard error
Ma et al. (2020)	China	cotton cloth vs. N95-typed	-	filtration efficiency	influenza aerosols	4/4	1.81	0.76
	China	cotton cloth vs. medical/surgical	-	filtration efficiency	influenza aerosols	4/4	0.63	0.63
Mueller et al. (2020)	USA	cotton cloth vs. medical/surgical	-	initial filtration efficiency	aerosols	9/9	1.78	0.54
	USA	cotton cloth vs. N95-typed	-	initial filtration efficiency	aerosols	9/6	6.98	1.37
Neupane et al. (2019)	Nepal	cotton cloth vs. medical/surgical	-	filtration efficiency	dust particles	12/3	2.55	0.77
Sharma et al. (2022)	UK	cotton cloth vs. N95-typed	-	filtration efficiency	aerosols	3/4	4.93	1.47
	UK	cotton cloth vs. medical/surgical	-	filtration efficiency	aerosols	3/3	2.61	1.00
Ueki et al. (2020)	USA	cotton cloth vs. N95-typed	gap	protective efficiency	viral RNAs	3/3	5.72	1.78
	USA	cotton cloth vs. N95-typed	no gap	protective efficiency	viral RNAs	3/3	14.17	4.14
	USA	cotton cloth vs. medical/surgical	-	protective efficiency	viral RNAs	3/3	-1.37	0.76
van der Sande et al. (2008) [d]	Netherlands	cotton cloth vs. N95-typed	-	protection factors	aerosols	7/8	1.05	0.52
	Netherlands	cotton cloth vs. medical/surgical	-	protection factors	aerosols	7/7	-0.90	0.53
Zhao et al. (2020)	USA	cotton cloth vs. medical/surgical	-	initial filtration efficiency	aerosols	6/6	7.70	1.66

Note. Positive effect sizes indicate better performance of non-cloth masks; [a] = means and standard deviations for effect size calculations were estimated based on median, minimum, and maximum values according to the approach of Luo et al. [22]; [b] = Single-layered cotton masks with 600 threads per inch were used for comparisons and filtration efficiency for particles < 300nm was assessed; [c] = Single-layered cotton masks with 600 threads per inch were used for comparisons and filtration efficiency for particles > 300nm was assessed; [d] = means and standard deviations for effect size calculations were estimated based on median and 25 as well as 75 percentile values according to the approach of Luo et al. [22].

Supplementary Figure 1

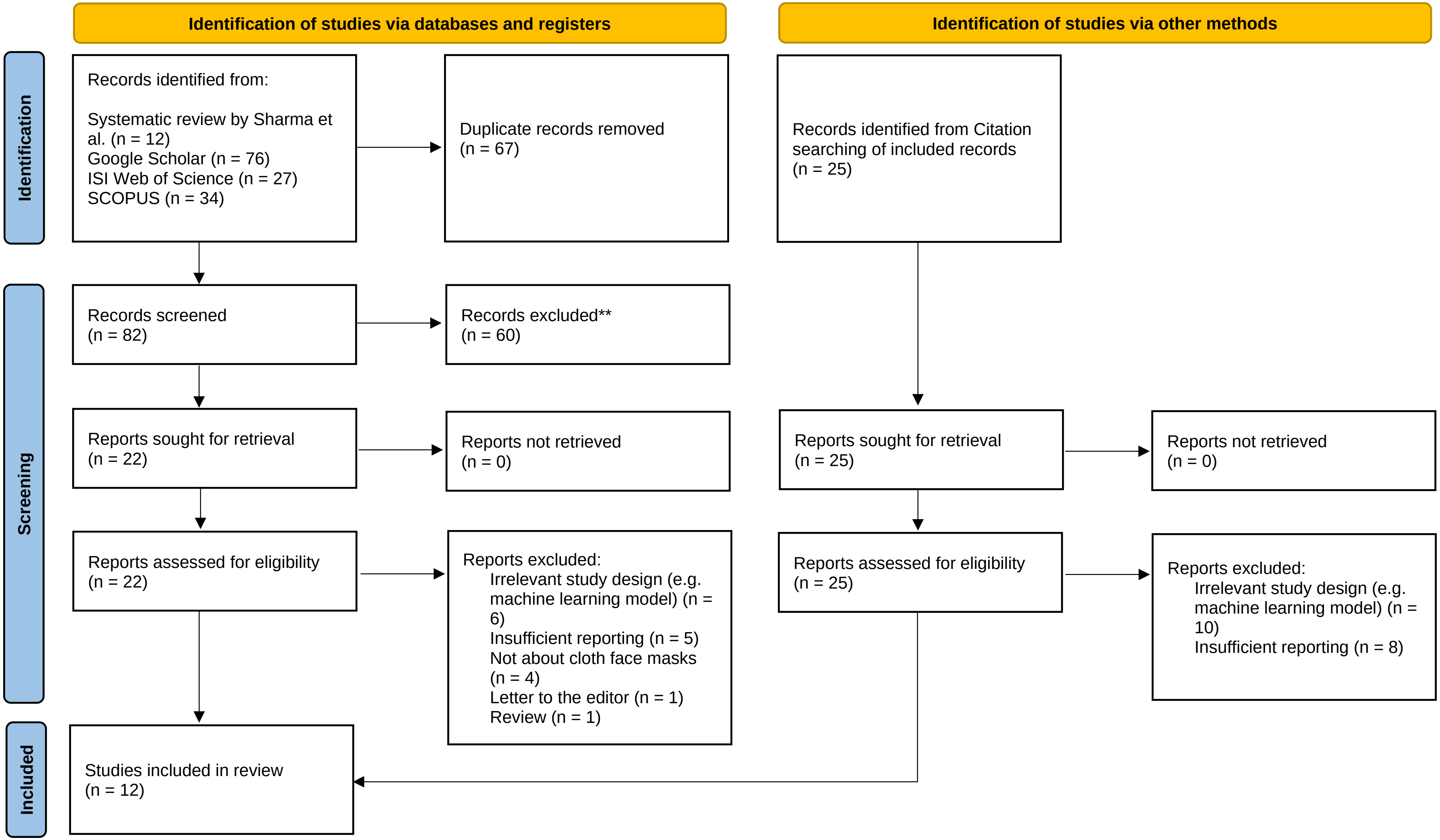

Supplementary Figure 2. Leave-one-out analyses for meta-analytical data (sub-)sets.

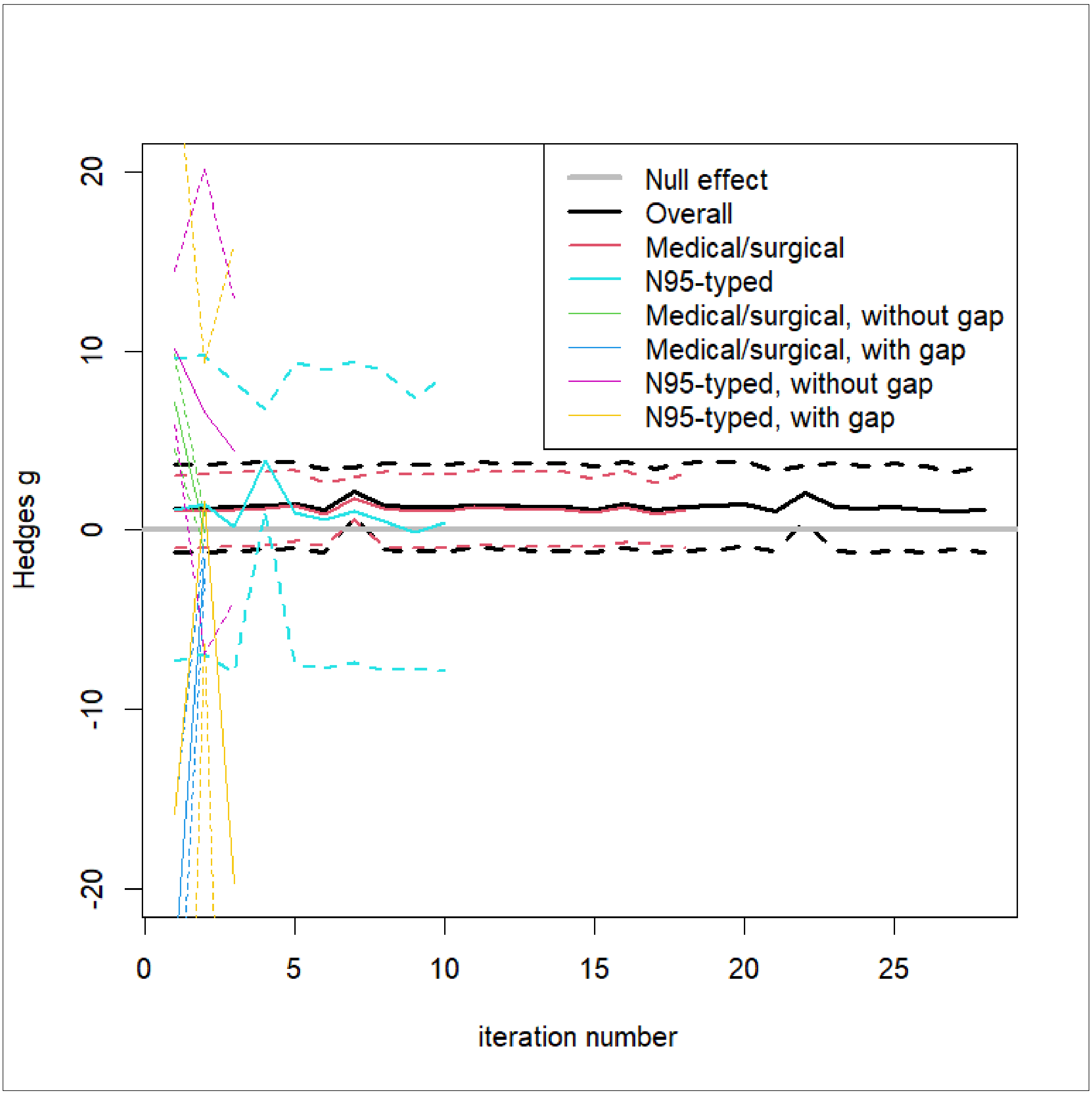

Note. Continuous lines represent changes in summary effects when individual studies are omitted from estimations; identically-colored dashed lines represent changes in associated 95% confidence intervals.

Supplementary File S1. References of papers included in analysis

[1] Aydin O, Emon B, Cheng S, Hong L, Chamorro LP, Saif MTA. Performance of fabrics for home-made masks against the spread of COVID-19 through droplets: A quantitative mechanistic study. Extreme Mech Lett 2020;40:100924. https://doi.org/10.1016/j.eml.2020.100924.

[2] Ayodeji OJ, Hilliard TA, Ramkumar S. Particle-size-dependent filtration efficiency, breathability, and flow resistance of face coverings and common household fabrics used for face masks during the COVID-19 pandemic. Int J Environ Res 2022;16:11. https://doi.org/10.1007/s41742-021-00390-6.

[3] Davies A, Thompson K-A, Giri K, Kafatos G, Walker J, Bennett A. Testing the efficacy of homemade masks: Would they protect in an influenza pandemic? Disaster Med Public Health Prep 2013;7:413–8. https://doi.org/10.1017/dmp.2013.43.

[4] Jung H, Kim JK, Lee S, Lee J, Kim J, Tsai P, et al. Comparison of filtration efficiency and pressure drop in anti-yellow sand masks, quarantine masks, medical masks, general masks, and handkerchiefs. Aerosol Air Qual Res 2014;14:991–1002. https://doi.org/10.4209/aaqr.2013.06.0201.

[5] Konda A, Prakash A, Moss GA, Schmoldt M, Grant GD, Guha S. Aerosol filtration efficiency of common fabrics used in respiratory cloth masks. ACS Nano 2020:0c03252. https://doi.org/10.1021/acsnano.0c03252.

[6] Ma Q, Shan H, Zhang H, Li G, Yang R, Chen J. Potential utilities of mask-wearing and instant hand hygiene for fighting SARS-CoV-2. J Med Virol 2020;92:1567–71. https://doi.org/10.1002/jmv.25805.

[7] Mueller AV, Eden MJ, Oakes JM, Bellini C, Fernandez LA. Quantitative method for comparative assessment of particle removal efficiency of fabric masks as alternatives to standard surgical masks for PPE. Matter 2020;3:950–62. https://doi.org/10.1016/j.matt.2020.07.006.

[8] Neupane BB, Mainali S, Sharma A, Giri B. Optical microscopic study of surface morphology and filtering efficiency of face masks. PeerJ 2019;7:e7142. https://doi.org/10.7717/peerj.7142.

[9] Sharma A, Omidvarborna H, Kumar P. Efficacy of facemasks in mitigating respiratory exposure to submicron aerosols. Journal of Hazardous Materials 2022;422:126783. https://doi.org/10.1016/j.jhazmat.2021.126783.

[10] Ueki H, Furusawa Y, Iwatsuki-Horimoto K, Imai M, Kabata H, Nishimura H, et al. Effectiveness of face masks in preventing airborne transmission of SARS-CoV-2. MSphere 2020;5:e00637-20. https://doi.org/10.1128/mSphere.00637-20.

[11] van der Sande M, Teunis P, Sabel R. Professional and home-made face masks reduce exposure to respiratory infections among the general population. PLoS One 2008;3:e2618. https://doi.org/10.1371/journal.pone.0002618.

[12] Zhao M, Liao L, Xiao W, Yu X, Wang H, Wang Q, et al. Household materials selection for homemade cloth face coverings and their filtration efficiency enhancement with triboelectric charging. Nano Lett 2020;20:5544–52. https://doi.org/10.1021/acs.nanolett.0c02211.
